# Bi incorporation and segregation in the MBE-grown GaAs-(Ga,Al)As-Ga(As,Bi) core–shell nanowires

**DOI:** 10.1038/s41598-022-09847-w

**Published:** 2022-04-09

**Authors:** Janusz Sadowski, Anna Kaleta, Serhii Kryvyi, Dorota Janaszko, Bogusława Kurowska, Marta Bilska, Tomasz Wojciechowski, Jarosław Z. Domagala, Ana M. Sanchez, Sławomir Kret

**Affiliations:** 1 Institute of Physics Polish Academy of Sciences, Aleja Lotnikow 32/46, 02668 Warsaw, Poland; 2 Department of Physics and Electrical Engineering, Linnaeus University, 39182 Kalmar, Sweden; 3 International Research Centre MagTop, Institute of Physics, Polish Academy of Sciences, Aleja Lotnikow 32/46, 02668 Warsaw, Poland; 4 Department of Physics, University of Warwick, Coventry, CV4 7AL UK

**Keywords:** Materials science, Physics

## Abstract

Incorporation of Bi into GaAs-(Ga,Al)As-Ga(As,Bi) core–shell nanowires grown by molecular beam epitaxy is studied with transmission electron microscopy. Nanowires are grown on GaAs(111)B substrates with Au-droplet assisted mode. Bi-doped shells are grown at low temperature (300 °C) with a close to stoichiometric Ga/As flux ratio. At low Bi fluxes, the Ga(As,Bi) shells are smooth, with Bi completely incorporated into the shells. Higher Bi fluxes (Bi/As flux ratio ~ 4%) led to partial segregation of Bi as droplets on the nanowires sidewalls, preferentially located at the nanowire segments with wurtzite structure. We demonstrate that such Bi droplets on the sidewalls act as catalysts for the growth of branches perpendicular to the GaAs trunks. Due to the tunability between zinc-blende and wurtzite polytypes by changing the nanowire growth conditions, this effect enables fabrication of branched nanowire architectures with branches generated from selected (wurtzite) nanowire segments.

## Introduction

Crystallization of Ga(As,Bi) ternary alloy is challenging since Bi acts as a surfactant during the epitaxial growth of III-V semiconductor layers^1^. However the application of dedicated thin film growth procedures allowed to obtain Ga(As,Bi) solid solutions with percentage range Bi content. Partial replacement of As anions by Bi heavy element leads to a substantial band-gap reduction and enhanced spin–orbit splitting in Ga(As,Bi), as compared with the binary GaAs host^2^. Ga(As,Bi) solid solution was first obtained by metalorganic vapor phase epitaxy (MOVPE)^3^, and then by molecular beam epitaxy (MBE)^4^. The latter became the most conventional method of growing Ga(As,Bi) thin films, since higher Bi contents were obtained than those achieved with the use of the MOVPE technique^5^. GaBi binary compound does not occur in the crystalline form^6^, and therefore, the amount of Bi atoms residing in the As lattice sites in the GaAs crystal is limited. The highest Bi concentration in Ga(As,Bi) reported so far is about 20%, obtained in thin layers grown by MBE at optimized conditions. A significant energy gap reduction, down to 0.5 eV, has been reported for 17.8% Bi content^7^. Hence Ga(As,Bi) bandgap can be tuned between that of binary GaAs (1.43 eV at room temperature) and 0.5 eV, making this material suitable for infrared optoelectronic applications^8^. Moreover, Ga(As,Bi) and other heavily Bi doped III-V semiconductors (e.g. antimonides) are expected to exhibit topological insulator properties^9^. These topological properties were first based on theoretical modelling, but they have recently been experimentally demonstrated for InBi binary compound hosting topologically protected surface states^10,11^. There is no similar experimental evidence of topological protection in Ga(As,Bi) yet, but this ternary alloy has not been investigated in this context so far. Recently topological properties of Bi-alloyed III-V semiconductors (arsenides and antimonides) in the wurtzite (WZ) polytype have also been theoretically predicted^12^. Since WZ III–V compounds can easily be obtained in the quasi 1D (NW) geometry^13^, the efforts to grow III–(V,Bi) ternary alloy NWs are interesting also in the context of topological materials. Although, Ga(As,Bi) planar layers grown by MBE have been investigated for over two decades, there is a very limited number of reports on Ga(As,Bi) in the NW geometry. The main reason is quite extreme MBE growth conditions of Ga(As,Bi) (in comparison with GaAs); i.e., low growth temperature (~ 200–350 °C) and close-to stoichiometric V/III elements flux ratio to avoid Bi surface segregation and to induce its substantial incorporation at As sites in the GaAs host lattice^14,15^. These growth conditions deviate from the GaAs NWs growth requirements, where higher substrate temperatures (> 500 °C) and high As excess are indispensable^16^. This implies that Ga(As,Bi) NWs with significant Bi content can only be obtained as low-temperature shells grown on Bi-free core NWs. This is similar to NWs implementing (Ga,Mn)As dilute ferromagnetic semiconductor with akin growth requirements, demanding even lower growth temperatures [~ 100 °C lower than Ga(As,Bi)] and the same stoichiometric III/V flux ratio^17–20^. Recently the mixed phase WZ-ZB GaAs NWs exposed ex-situ to Bi vapor were studied by scanning tunneling microscopy^21^ but this study has little reference to our investigations of in-situ Bi incorporation.

In this paper we thoroughly investigate the radial distribution of Bi in GaAs NWs (not studied so far to our knowledge) and elucidate the emergence of side Bi droplets and branches on the wurtzite segments of GaAs NWs with Ga(As,Bi) shells.

## Samples and experimental methods

The core–shell nanowires were grown in a dedicated III–V MBE system. First the GaAs NW trunks were crystallized on GaAs(111)B substrates by the Au-assisted growth mode. 5 Å thick gold film was deposited on epi-ready GaAs(111)B wafers in another MBE system, and transferred (in air) to the III–V one. The growth was monitored by reflection high energy electron diffraction (RHEED) system. The substrate temperature was controlled by the MBE substrate manipulator thermocouple, calibrated using GaAs(100) surface reconstruction transition temperatures^22^. For that, a piece of GaAs(100) wafer was placed in the vicinity of Au-coated GaAs(111)B dedicated to the NWs growth. Planar Ga(As,Bi) layer grown on GaAs(100) during the Ga(As,Bi) NW shells deposition also serves as references to compare Bi incorporation into ZB and WZ GaAs phase of planar layers and NW shells, respectively (see the Supplementary Information). After preheating the substrates to 600 °C in the MBE growth chamber resulting in the thermal desorption of native oxide and formation of AuGa eutectic droplets at random surface sites of GaAs(111)B wafer, the substrate temperature was decreased to 540 °C, and GaAs NWs have been grown for 1–3 h, depending on the sample. The Ga flux intensity (calibrated through a test growth on GaAs(100) substrate by RHEED intensity oscillations) corresponded to the planar growth rate of 0.2 μm/h, which resulted in the axial NWs growth rate of about 1 μm/h. As the source of arsenic, the valved cracker cell has been used with cracking zone temperature of 950 °C i.e., As dimers were prevailing in the As flux. The As/Ga flux ratio during this growth stage was about 10. The growth of GaAs NW trunks was completed by closing Ga and As shutters. The latter was closed one minute after the first one. After closing the Ga shutter the substrate temperature was decreased for deposition of the NW shells. In the case of one sample (sample 3) prior to the Ga(As,Bi) growth, about 30 nm (Ga,Al)As shells have been grown at the substrate temperature of 400 °C, with As and Ga flux ratios the same as used previously for the axial GaAs NWs growth. For the deposition of Ga(As,Bi) shells the substrate temperature was further decreased to about 300 °C. Ga(As,Bi) shells have been deposited in close-to-stoichiometric growth conditions i.e. with V/III flux intensity ratio close to 1.

We investigate three different types of GaAs-Ga(As,Bi) NW samples. The Ga(As,Bi) shells were grown in similar conditions for all the samples, i.e. at the same substrate temperature (300 °C) and As/Ga flux ratio (~ 1). The main difference between the growths was the Bi flux intensity during deposition of the low temperature (LT) Ga(As,Bi) shells. Bi flux was generated from a standard Knudsen effusion cell. To obtain different Bi concentrations the Bi cell temperature was set to 540 °C or 580 °C, corresponding to low (1%) or high (2–4%) Bi content in Ga(As,Bi) shells of sample 1, (2 and 3), respectively. In each case the Ga(As,Bi) shell was finished by the deposition of 4–7 nm thick LT GaAs.

**Figure 1:**
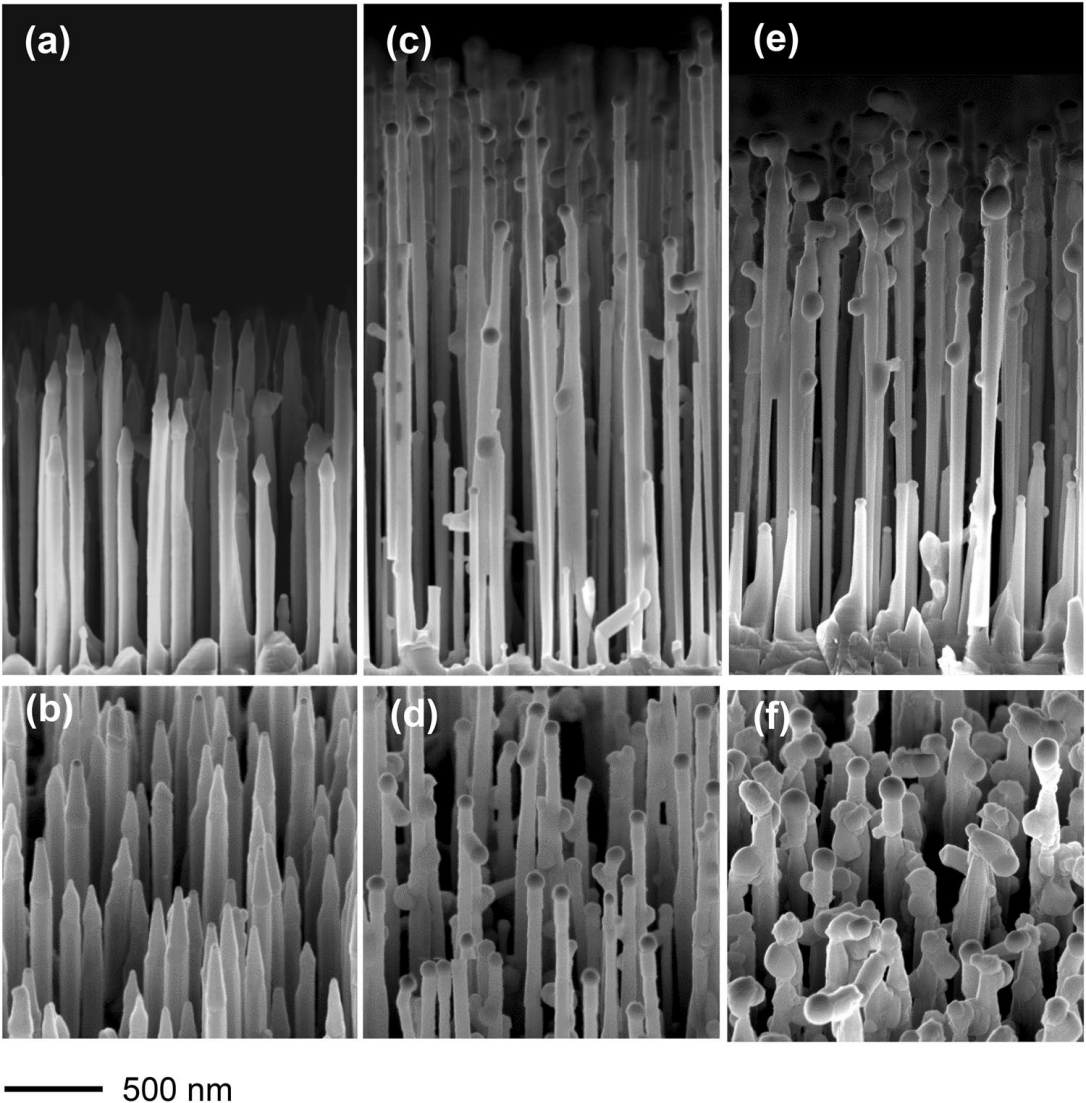
SEM images of GaAs-Ga(As,Bi) core–shell nanowires grown on GaAs(111)B substrate. (**a**,**b**) sample 1 (1% Bi); (**c**,**d**) sample 2 (4% Bi); (**e**,**f**) sample 3 (4% Bi and additional (Ga,Al)As shell in-between GaAs NW trunk and Ga(As,Bi) shell. Upper panels—cross-sectional views, lower panels—45° tilted views. The 500 nm scale bar plotted in the bottom part of the figure is common for all the panels.

Figure 1 shows the scanning electron microscopy (SEM) images of samples 1, 2 and 3 grown with low (sample 1) and high Bi flux (samples 2, and 3), at the Bi effusion cell temperature (T_Bi_) of 540 °C and 580 °C, respectively. NW sidewalls in sample 1 have no side droplets. The conical features at the NW tips (with clearly visible Au droplet at the very top) are due to the residual axial growth during the Ga(As,Bi) shell deposition at 300 °C. In samples 2 and 3 grown with T_Bi_ = 580 °C most of the NWs have side droplets, and short branches perpendicular to the main NW trunks.

A deeper insight into the NWs structure has been obtained by transmission electron microscopy (TEM) investigations. Morphology and structure of the NWs have been investigated using a FEI Titan 80–300 transmission electron microscope (TEM) operating at 300 kV with a spherical aberration corrector of objective lenses in the HRTEM mode and a doubly corrected ARM200F microscope, operating at 200 kV. The elemental composition determination was carried out using EDAX 30 mm^2^ Si(Li) detector with a collection angle of 0.13 srad and a 100 mm^2^ Oxford Instruments windowless EDX detector installed within the Jeol ARM200F microscope.

For TEM investigation the NWs have been transferred mechanically onto copper grids covered with holey carbon films enabling imaging the entire NWs. The cross-sections of the epoxy resin or platinum–carbon composite embedded NWs have been cut using a focused ion beam in Helios Nanolab 600 FIB as it is described in Ref.^23^.

## Results and discussion

**Figure 2:**
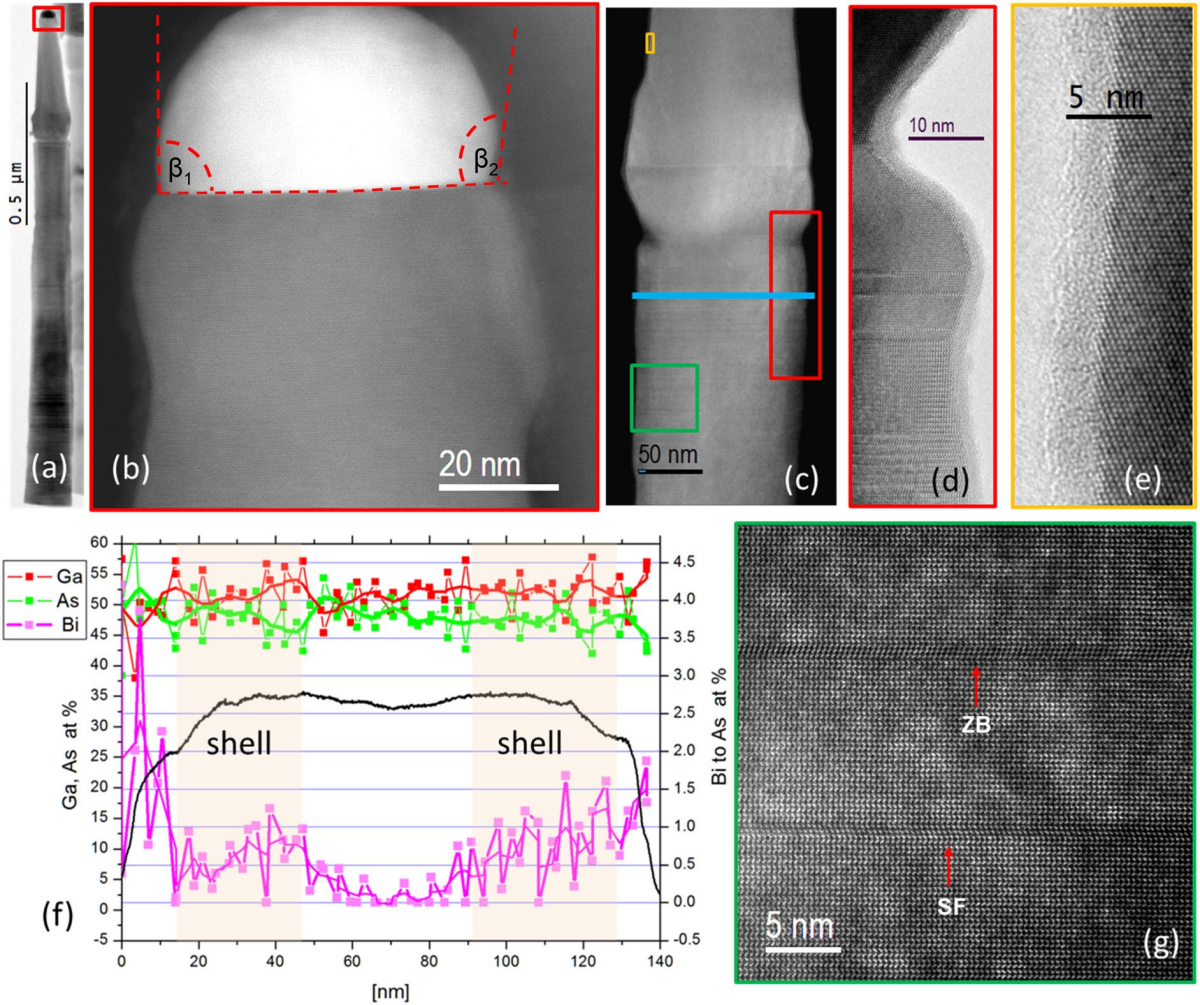
(**a**) TEM image of a GaAs-GaAs_0.99_Bi_0.01_ core–shell nanowire (sample 1), conical tip results from the residual axial growth during Ga(As,Bi) shell deposition; (**b**) ADF-STEM image of the upper zinc-blende stacking fault free part of NW below gold catalyzer droplet; (**c**) STEM image of the transition part of the NW between GaAs core and the section which grows during Ga(As,Bi) shell deposition; (**d**) HRTEM image showing progressive transition from WZ to pure ZB structure of the top NW part displayed in (**e**)—marked as small yellow rectangle in (**c**); (**f**) EDS elemental distribution profile for As, Bi and Ga along the blue line together with the STEM intensity; (**g**) typical structure of the bottom part of the NW bellow the transition zone evidencing mainly WZ structure, the single SF and the 3 ML thick ZB inclusion are also visible.

Figure 2 shows TEM and scanning transmission electron microscopy (STEM) images of an individual NW from sample 1, grown with low Bi flux. Interestingly, pure ZB structure was revealed at the conical NW tip (Fig. 2b,e). No distinct lines revealing stacking faults (SF) or twin boundaries (TB) are visible. We infer that these ZB tips emerge due to the residual axial growth during Ga(As,Bi) shell deposition. The pure ZB phase at the upper NW section is consistent with the reported dependence of GaAs NW phase on the NW tip–droplet contact angle^24^. The authors of Ref.^24^ observed three different regions of droplet–NW tip contact angle ranges (here defined as β) inducing WZ or ZB structure during the NW growth. For low β values (80–100 deg.), the ZB phase is promoted, intermediate β values (100–125 deg.) promote WZ phase, and higher β values (125–140 deg.) again induce the occurrence of ZB phase in the NW. As can be observed in Fig. 2b, the droplet contact angles for the NW tip are in the low β range, 90° on the left and 100° on the right side of the NW-droplet interface (β_1_ and β_2_, respectively), which according to Ref.^24^ should promote ZB phase of GaAs NWs.

The Bi content values exceeding 1at.% at the surface of NWs (see the STEM intensity profile in Fig. 2f), are the quantification artefacts. Quantification is based on the Bi M-line of the intensity clearly above the noise level in the spectrum. Also the As concentration drops to 99% with corresponding 1% Bi content detected. Quantification based on Bi L-line gives higher values of about 2 at.% but this line coincides with the As-K peak energy which can increase the apparent bismuth content due to not perfect deconvolution. The elemental distribution shown in Fig. 2f is expressed in at.%, for Ga and As, whereas the Bi concentration is normalized to the As atoms. From the Bi distribution and STEM-HAADF profile we can estimate the NW core diameter of ~ 40 nm and the Ga(As,Bi) shell thickness of ~ 30–40 nm. The maximum concentration of Bi is found at the NW side-wall surface (the amorphous “skin” of the NW—see Fig. S5 in the Supplementary Material), but the maximum concentration of Bi/As in the shell is at the level of ~ 1%. Interestingly in the planar ZB Ga(As,Bi) layer grown together with sample 1, the Bi content evaluated from the Ga(As,Bi) lattice parameter (see Fig. S1 in the supplementary material) amounts to 4.6%, which proves much effective incorporation of Bi into planar ZB GaAs(100) than to WZ GaAs(11–20) NW sidewall planes.

**Figure 3:**
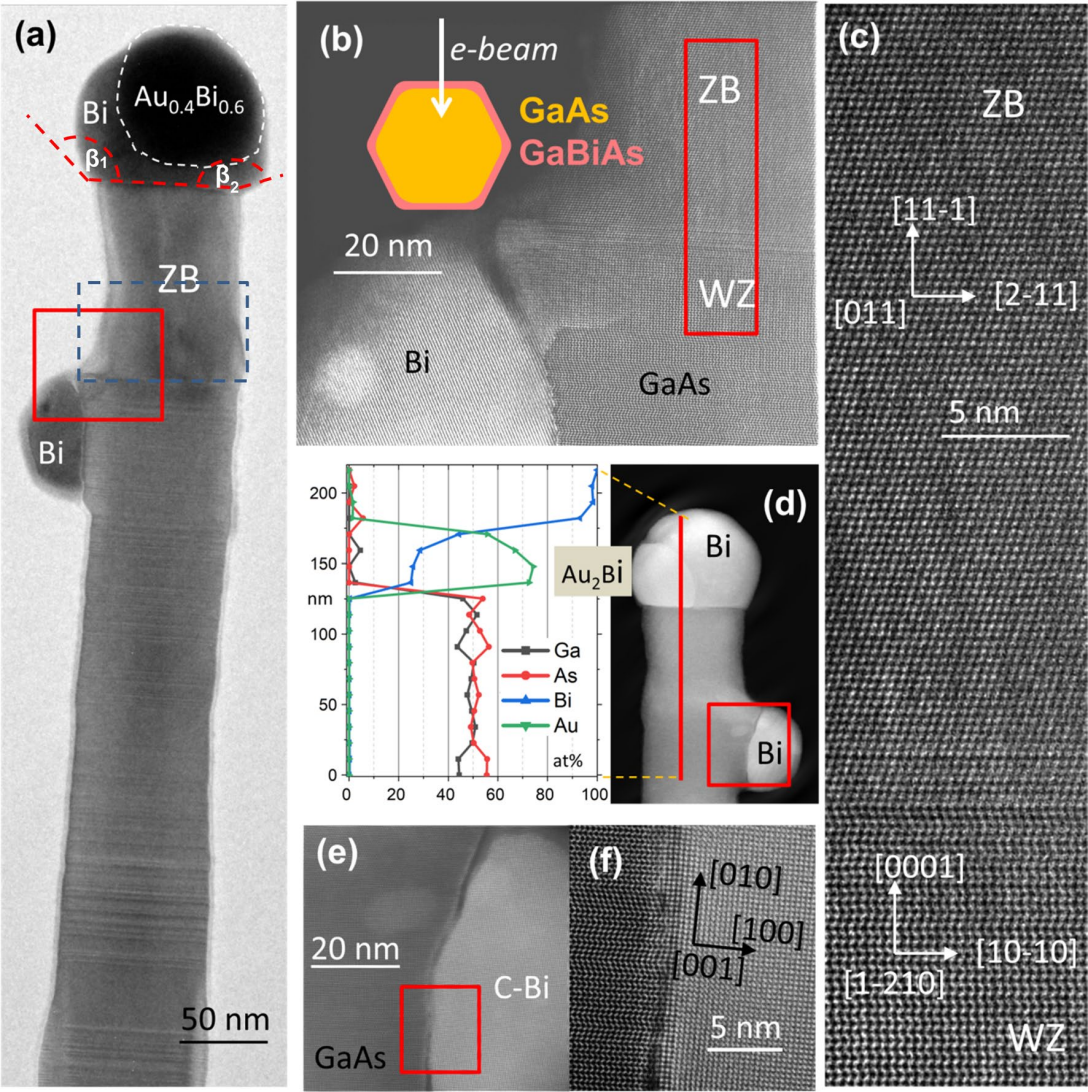
(**a**) HRTEM of a NW from sample 2; (**b**) STEM of the frame in (**a**), a diagram shows orientation of the facets in relation to the e-beam; (**c**) STEM of the frame in (**b**); (**d**) STEM image of another NW and EDS elemental concentration profile along the red line; (**e**) STEM zoomed part of the frame from (**d**); (**f**) zoomed part of the frame marked in (**e**).

In order to increase the Bi content in Ga(As,Bi) NW shells, sample 2 was grown with much higher Bi flux intensity, than that used for sample 1. SEM images of sample 2 (Fig. 1c,d) show longer NWs in comparison to sample 1 (Fig. 1a,b). GaAs cores in sample 2 were grown for longer time (3 h 15 min vs 1 h growth time relevant for sample 1), hence the NW lengths reach up to 4.5 μm. The Ga(As,Bi) shells were grown with T_Bi_ = 580 °C which corresponds to ~ 4 times higher Bi flux (sample 1 was grown with T_Bi_ = 540 °C). The detailed TEM analyses of the NW collected from sample 2 are shown in Fig. 3. The main difference between samples 2 and 1 is the occurrence of droplets at the NW sidewalls in the former—see Figs. 1c,d; 3a,d. Apparently, the Bi excess, which is not incorporated into Ga(As,Bi) shells, accumulates as droplets at the NW sidewalls. Additionally, a thin amorphous Bi layer can be observed at the NW sidewalls (Fig. S5 in the Supplementary Material). Moreover, as shown in Fig. 3d, a substantial Bi content is also revealed in the catalytic droplet at the NW top (solidified into nanocrystal at room temperature TEM investigations). The top droplet clearly consists of the two distinct parts. The part appearing as brighter seems to be embedded in the bigger, darker one (see Fig. 3d). The EDS analysis shows that the larger droplet consists of pure, solidified Bi. The elemental composition of the smaller part suggests that it contains a metastable Au_~0.40_Bi_~0.60_ phase^6^. The side droplet shown in Fig, 3a,b contains mainly Bi with a small admixture of As (up to 3 at.%). This droplet crystallized just below the transition region from mainly WZ to the pure ZB part. The details of this transition zone are shown in Fig. 3b,c. This WZ-ZB transition is quite abrupt, along about 5 nm long NW section. The upper ZB part is free  from SF or TB defects. Figure 3d shows another NW also containing top and side droplet. Similarly to the first NW shown in Fig. 3a,b, the top droplet also consists of two parts: pure Bi and Au-rich Au_2_Bi phase. Also in this case, the pure ZB structure part is about 100 nm long. The details of this part are shown in Fig. S4 of the supplementary material, revealing that it hosts an oblique SF which starts at the WZ-ZB transition zone and ends at Au_2_Bi/Bi interface. This suggests that Au_2_Bi phase nucleated at the SF and emerged during residual axial growth of this upper (ZB) NW part. Hence this part grew in a dual VLS and (vapor–solid-solid) VSS mechanism.

The side Bi droplet crystallized in the uncommon cubic Bi phase^25^ with the lattice parameter of 3.17 Å (see Fig. 3f). The (010) planes in the Bi droplet are in the epitaxial relation to WZ GaAs planes d_(0002)_. The Bi lattice planes in vertical direction inside the droplet visible in STEM images (Fig. 3f) have the interplanar distance d_(010)_ = 3.285 Å but in the radial direction the spacing d_(100)_ equals to 3.33 Å. This means that the measured lattice parameter is about 3% bigger than that reported in Ref.^25^ and we also detect a tetragonal distortion of 1.7%. The lattice planes of Bi in the (solidified) side droplets are rotated clockwise by 1 deg. with respect to the core WZ GaAs lattice.

In contrast to sample 1 with pure top Au droplets with diameters of ~ 55 nm and 400 nm long, the conical ZB parts below the top droplet of sample 2, (see Fig. 3a) are much larger in diameter—about 100 nm, but the lengths of the top ZB segments are much shorter—about 110 nm. From now on, we will assign the name *“NW neck”* to the top ZB segment of the NW, which results from the residual axial growth during the low-temperature Ga(As,Bi) shell deposition (as discussed above)*.* The shape differences of the NW necks in samples 1 and 2 can be explained as follows. In both cases, two growth modes of the NW neck occur simultaneously: (i) axial growth and (ii) radial growth. In sample 1, the catalytic droplet is small, without any Bi, since the Bi flux is low and all impinging Bi is incorporated into the NW shells (see Figs. 1a,b and 2a). It is well-known that when the growth is controlled by the diffusion flux of adatoms along the NW sidewalls towards the top, the axial NW growth rate is inversely proportional to the size of the catalytic droplet^26^. Smaller droplets in sample 1 induce faster residual axial growth during the Ga(As,Bi) shell deposition. The shell growth time in sample 1 was 1 h, resulting in 400 nm long conical ZB NW neck. The Ga flux applied during the shell growth corresponded to the high temperature axial growth rate of the GaAs NW trunk equal to 1 μm/h. The axial growth rate of the NW neck during the 1 h shell deposition is 2.5 times slower due to the low temperature of the shell growth (about 300 °C), and the competing mechanism of the radial growth. The conical shapes are typical for NWs grown at low temperatures, where the axial and radial growth modes occur simultaneously^16^. Here, the top droplet is ~ 100 nm in diameter; twice the size of the droplet in sample 1, but the ZB NW neck is only ~ 120 nm long (in contrast to 400 nm long neck of sample 1). The shell deposition time for sample 2 was 0.5 h (1 h for sample 1), with the same Ga flux intensity, corresponding to a planar growth rate of 0.2 μm/h. The different neck shape in Sample 2 (in comparison to Sample 1) is most probably correlated to the larger top droplet size upon exposure to high Bi flux during the Ga(As,Bi) shell growth, with the top droplet gradually accumulating more Bi, which compensates the tapered NW shape (even reversed tapering can be inferred from Fig. 3a induced by the low growth temperature). Standard NW tapering was observed in the first 50 nm of the NW neck (see section enclosed by the blue dashed rectangle in Fig. 3a), whereas a slightly inverse tapering is visible in the section above. The common tapering effect associated with the radial growth, most pronounced at the lower part of the NW neck was compensated by the increasing diameter of the upper neck part, due to increasing size and Bi content of the top droplet. This effect can be observed in the two different NWs shown in Fig. 3a,d. The measured droplet contact angles β_1_ and β_2_ are equal to 135 and 200 deg. respectively in the NW tip—NW top droplet section shown in Fig. 3a. According to the in-situ TEM NW growth investigations reported in Ref.^24^, the catalyzing droplet—NW tip contact angles higher than about 125 deg. promote the ZB GaAs NW phase.

**Figure 4:**
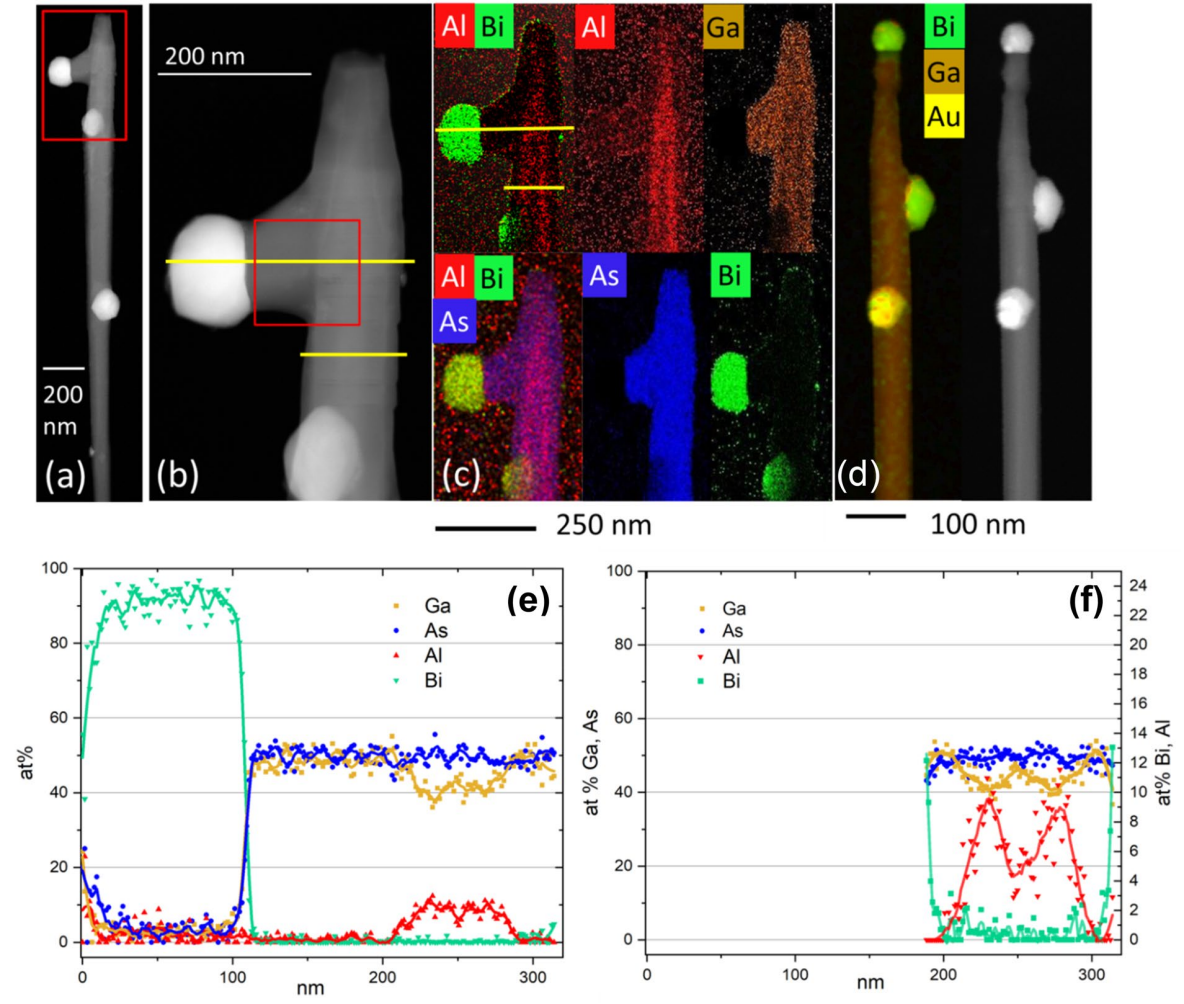
(**a**,**b**) TEM images of the upper GaAs-(Ga,Al)As-Ga(As,Bi) core-double shell NW part with Bi-catalyzed side branch (the very top part with the top catalyzing droplet has been broken during the NW collection), the side Bi droplet catalyzes the branch growth; (**c**) EDS composition maps visualizing the distribution of the elements: Ga, Al, As, Bi in the NW section shown in (**b**); (**d**)—EDS composition map of another NW picked from Sample 3, showing Au droplet, which slid downwards and was replaced by the Bi droplet at the NW top; (**e**,**f**)—EDS line scans showing the distribution of the elements along yellow lines marked in (**b**,**c**).

To get better insight into the formation and morphology of Ga(As,Bi) shells, a ~ 15 nm thick (Ga,Al)As shells, with 30% Al, had been grown on the GaAs NW core before the Ga(As,Bi) shell growth, in the case of sample 3. The (Ga,Al)As shells were deposited at 400 °C. The lighter (Ga,Al)As layer contrast in ADF-STEM images (see Fig. 4b) allows to unequivocally identify the Ga(As,Bi) shells both in the plan-view and cross sections images of the NW. EDS compositional distribution across the WZ area of the NW shown in Fig. 4f reveals that the NW shell contains about 1at. % Bi.

The Ga(As,Bi) shells were grown in the same conditions (growth temperature, Bi and Ga fluxes, As/Ga flux ratio) as sample 2, but for a slightly longer time (45 min for sample 3, versus 30 min for sample 2). The 50% longer Ga(As,Bi) shell growth time under Bi excess conditions leads to a more pronounced Bi segregation forming more droplets at the NW sidewalls. Figure 4 summarizes the compositional analysis carried out for two representative NWs from sample 3. Two different upper GaAs-(Ga,Al)As-Ga(As,Bi) core-double shell NWs are displayed. The first one shown in Fig. 4a–c has a missing top. The HRSTEM images of the Bi droplet catalyzed side branch and branch-trunk regions are shown in Fig.S6 of the Supporting Information. Similarly to the case of sample 2 the residual axial growth during crystallization of Ga(As,Bi) shell produces distinct necks (visible also in Fig. 1e,f). This neck part can easily be broken during the mechanical transfer of NWs to the TEM grid. A complete NW is shown in Fig. 4 d and Fig. S7 in the Supporting Information. The EDS maps (Fig. 4c) and profiles (Fig. 4e,f) reveal the inner GaAs core and (Ga,Al)As shell. This data let us conclude that during the growth of (Ga,Al)As shell also axial (Ga,Al)As growth was continued over the length of ~ 200 nm. After the temperature drop necessary to grow Ga(As,Bi) shell, the gold droplet was replaced by the Bi one, and axial growth continued in both axial and slightly off-axis (random) directions, as can be seen in Fig. 1e,f.

The accumulation of liquid Bi at the sidewall surface resulted in the formation of additional Bi-rich droplets, which started to catalyze the secondary branches reproducing the trunk structure—twinning ZB parts and SF in WZ parts of the core, as shown in Fig.S6 in the supporting information. Replacing the Au droplet by the Bi one at the NW top can be explained as follows. The temperature decrease to 300 °C for Ga(As,Bi) shell deposition caused crystallization of Au at the NW top, but after delivery of Bi in large amount, the external part of hitherto crystalized Au droplet became liquid. In the Au-Bi system, there are two eutectic points 371 °C and 241 °C, depending on the Bi concentration^6^. We infer that the 241 °C BiAu eutectic was formed and the surface of the gold nanocrystal at the NW top became liquid, which allowed the droplet to move and float down as shown in the EDS map in Fig. 4d. High magnification ADF-STEM image of the Bi-droplet catalyzed branch, corresponding to the region marked by the red square in Fig. 4b, is shown in Fig.S6 in the Supporting Information.

**Figure 5:**
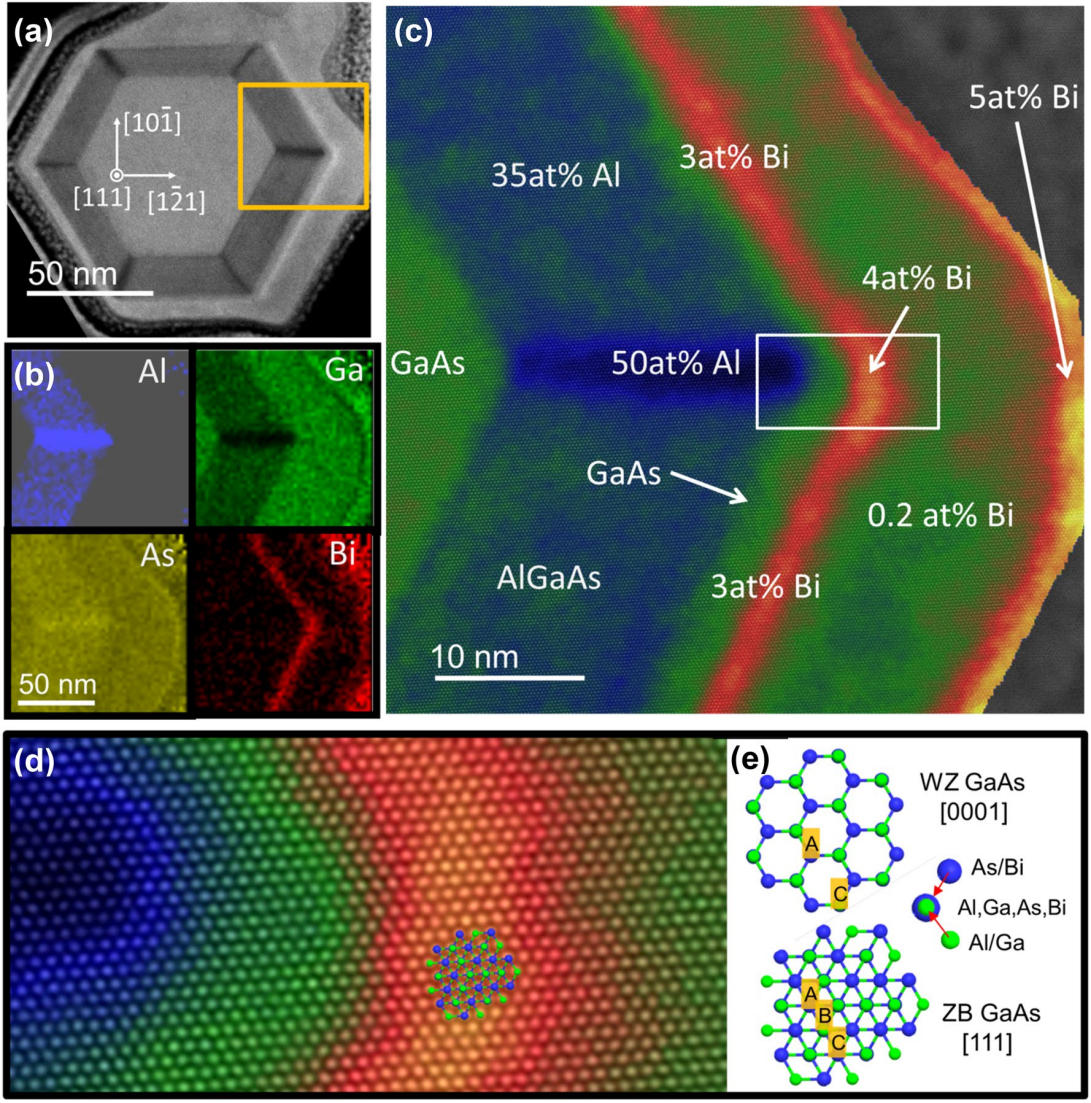
Analysis of a thin cross-section of ZB GaAs-(Ga,Al)As-Ga(As,Bi) NW segment. (**a**) ADF-STEM image of the entire NW cross-section, the yellow frame indicates the area of EDS maps; (**b**) EDS distribution maps of individual elements (Al, Ga, As, Bi); (**c**) result of the semi-quantitative EDS analysis displaying Al, Ga and Bi distribution. Different colors and their saturation correspond to different composition of Ga, Al and Bi (green, blue and red, respectively) determined on the basis of the EDS signal and STEM intensity; (**d**) zoomed part of frame from (**c**) with superimposed model of ZB sees towards < 111 > ; (**e**) comparison of WZ and ZB structure.

Figure 5 shows STEM images and EDS composition maps of a 75 nm thick FIB cross-section of the ZB fragment of GaAs-(Ga,Al)As-Ga(As,Bi) NW (sample 3). High magnification ADF-STEM images are obtained using a HAADF detector with angular collection between α_min_ = 65 mrad and α_max_ = 210 mrad. The thickness of the analyzed NW cross-section is measured using position averaged convergent beam electron diffraction (PACBED) technique as described in the Supporting Information. Under these recording conditions, the HAADF images are sensitive to the average atomic number (Z) of the atom column (see Fig. 5a). As can be seen in Fig. 5a the hexagonal shape of the GaAs core is reproduced in the successive shells. However, the last LT-GaAs outer shell does not develop sharp corners. The measured thicknesses of the (Ga,Al)As shells on all six NW sidewalls are equal to 18–20 nm.

The dark lines running from the GaAs core corners visible in Fig. 5a have already been reported^27–29^ for ZB NWs with (Ga,Al)As shells where high Al concentration, i.e. ~ 50% was detected in these regions and 30% Al elsewhere. However the Bi composition profile in the NWs with Ga(As,Bi) shells was not investigated so far, since no GaAs-Ga(As,Bi) NW cross-sections were studied before, to our knowledge. At the (Ga,Al)As-Ga(As,Bi) shell interface, the very thin adjacent Ga(As,Bi) shell region with higher Bi concentration appears as brighter lines in Fig. 5a (red stripes in Fig. 5b,c). Moreover, additional, very thin bright lines are visible at the very edge of the NW (orange in Fig. 5c). It should be noted that the Ga(As,Bi) shell thicknesses are slightly different at each NW sidewall especially for the sidewalls in the closest neighborhood to other NWs, due to the shadowing effect during the MBE growth. Figure 5b shows the EDS elemental composition analysis of the cross-section specimen. The analysis was performed for the part enclosed by the yellow rectangle in Fig. 5a. The data presented in this Figure evidence direct correlation between the STEM image intensity and the concentration of elements visualized in the EDS maps as explained below.

The average intensity level of the STEM image in the GaAs core region amounts to 15,800 counts per pixel what we consider as a reference value 1. STEM intensity reaches maximum of 19,400 counts/pixel (1.23 in this convention) in the corners of hexagon which also corresponds to the maximum Bi concentration obtained from the EDS measurement. Moreover, in the areas of characteristic dark contrast (the features lying along the < 112 > crystallographic direction) the STEM intensity drops to 0.63.

Figure 5c shows a false-colored HR-STEM image obtained by combining information from EDS maps and intensities of STEM images. The STEM intensity was scaled based on the EDS results in the following way: in the case of Al and Ga the average ratio of Al/Ga from the shell region and from high Al content area were referred to the average STEM intensity in the same area. Then, the linear dependence of Al/Ga and STEM intensity was assumed. In the case of Bi, a similar procedure was applied but the average values for the middle of the Bi rich area were taken into account. For calculating the average chemical composition in the given area, the sum of spectra was used for elements quantification. Finally, the elemental composition maps were plotted with higher spatial resolution than original EDS maps of 50 × 50 pixels. The average Al concentration in the (Ga,Al)As shell is at the level of 30 at.% with respect to Ga. The maximum concentration of 50 at.% Al is reached at the corners of the (Ga,Al)As shell. The average concentration of Bi with respect to As in the NW cross-section reaches 3 at.% but it is higher (4 at.%) at the corners of the hexagon. The highest Bi concentration (5 at.%) is measured at the NW sidewall surface, however some Bi atoms are not embedded into GaAs lattice but remain at the surface as a very thin amorphous layer. The average concentration of Bi between the Bi rich layer close to the inner (Ga,Al)As shells and the sidewall surface drops to small values generally less than 1 at. % (green areas in Fig, 5c). However the Bi content in the areas with Bi concentration smaller than ~ 0.2 at% which is in our case under the detection limit (blue areas in green-colored Ga(As,Bi) shell) is only roughly estimated, however the intensity of Bi-M line in the average spectrum is still above the noise level. Additionally, between the (Ga,Al)As shell and Bi-rich shell, the pure GaAs region with a thickness ~ 1–2 nm is detected. Such a thin GaAs shell was intentionally deposited to avoid (Ga,Al)As surface contamination during the relatively long growth break required to stabilize a low temperature of Ga(As,Bi) shell deposition (300 °C) as well as to decrease and stabilize As_2_ flux to close-to stoichiometric value to the Ga one. On other hand, the presence of such a layer clearly indicates that Bi bulk diffusion does not occur during the LT growth of the Ga(As,Bi) shell. The perimetric fluctuations of Bi concentration in the shell in the 2–5 nm scale are visible in Fig. 5c. However, averaging the information over 70–80 nm thick specimen blurs the localized Bi concentration fluctuations or clustering. Figure 5d shows atomic resolution STEM image of the area marked with the white rectangle in Fig. 5c, visualizing (Ga,Al)As-Ga(As,Bi) inner shell interface. The arrangement of atoms unequivocally points on the ZB structure of this NW slice. This is confirmed by the relevant ZB crystal structure model, shown in Fig. 5e and superimposed on the atomic resolution STEM image (Fig. 5d).

In Fig. 5e, A, B, C refer to three positions of the cation/anion columns bonded in the ZB < 111 > or WZ < 0001 > direction (i.e. along the cation–anion bonding in the direction of tetrahedra heights). For a perfect crystal, without SFs, all the atom columns observed in the [111] or [0001] zone axis (ZB or WZ case, respectively) should correspond to the local maxima with the same intensities in STEM images, so they are indistinguishable. However, in the presence of SF in the WZ structure, contrast maxima in positions B can appear in the STEM image and local maxima can differ in intensities, which makes it possible to distinguish between ZB and WZ phase in a thin cross-section specimen.

**Figure 6:**
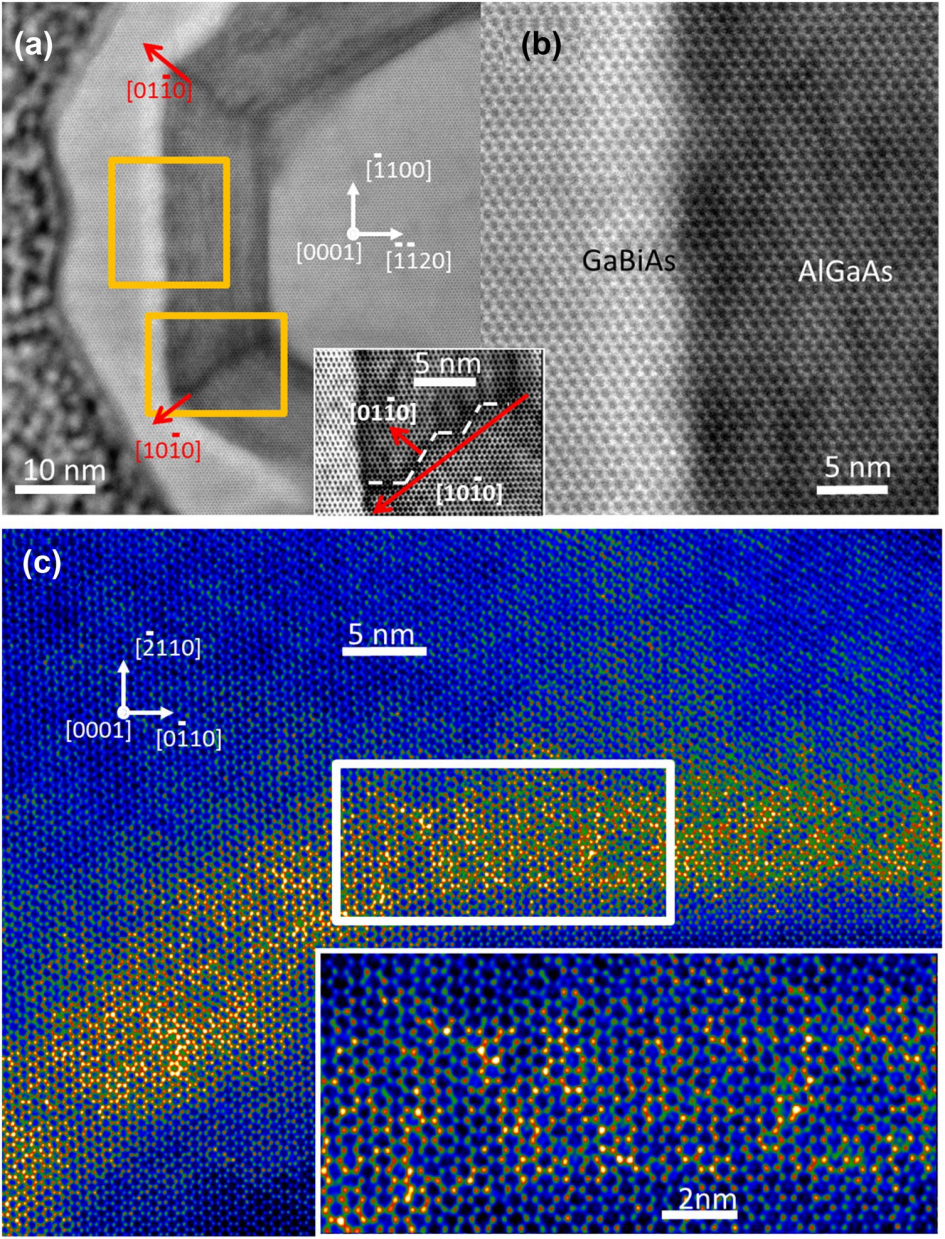
The analysis of a thin cross-section of the WZ GaAs-(Ga,Al)As-Ga(As,Bi) NW segment. (**a**) HR-STEM image acquired with the camera length of 73 mm; (**b**) zoomed part of the interface between (Ga,Al)As and Ga(As,Bi) shells; the inset to (**a**) and (**b**)—zoomed filtered image showing details of Al segregation at the NW corner; (**c**) HR-STEM (in false colors) of about 20 nm thick cross-section of Bi-rich shell; yellow color represents Bi-rich atomic columns averaged over 20-nm thick specimen, the inset shows zoomed area enclosed by the white rectangle.

Figure 6 shows a cross-sectional TEM image along the [0001] direction recorded in the WZ section of a NW from sample 3. In this case, the WZ phase can be easily identified due to fact that the only contrast maxima corresponding to …ACAC… stacking are visible, contrary to ZB part where maxima corresponding to ..ABC… stacking are visible (compare Fig. 5). In this case, the hexagonal shape of the core is also preserved. More detailed discussion concerning distinctiveness of ZB and WZ phases in the TEM cross-section specimen is included in the supplementary material. The 5–6 nm thick Bi-rich shell can be unequivocally identified in Fig. 6. This shell appears homogenous for all three NW facets visible in the image. We estimate that the specimen thickness is about 90 nm, so the fine Bi fluctuations cannot be detected. The segregation of Al is clearly visible inside the (Ga,Al)As shell. We also detect Al segregation at the edges of the hexagon, never reported before for WZ NWs. However, the segregation shown in the inset to Fig. 6a,b looks differently than that occurring in ZB (Ga,Al)As NW sections, reported earlier^27^. In our case, the high Al concentration regions have zigzag shapes^27^. Slightly similar zig-zag corner-line shapes were observed for P-rich regions of ZB Ga(As,P)/GaAs coaxial NWs^30^. The structural details of this Al-enhanced region are shown in the filtered image (inset to Fig. 6) where the zigzag shape, Al-rich path (dashed line) does not follow solely radial^10^ direction but consists of two crystallographic directions^10^ and^1–10^. This difference, with respect to the (Ga,Al)As NW shell of ZB structure is related to the lower symmetry of WZ structure, where the diffusion paths can be limited in comparison to the ZB one. The WZ NW cross-section shown in Fig. 6c is the thinnest specimen prepared with the FIB technique without its significant amorphization. We estimate the thickness of this cross-section to be less than 20 nm. Hence the local fluctuations of Bi atoms start to be visible with STEM and are much higher than those in the Bi-poor LT-GaAs shell. The Bi-rich areas are generally random but some texture in the radial direction can be distinguished; such areas with the diameters of 1–2 nm can be seen. In the cross-sections of both WZ and ZB parts of a NW collected from sample 3, we notice inhomogeneous concentration of Bi in the radial direction. Similar Bi concentration profile was also observed in the MBE-grown planar Ga(As,Bi) layers, with Bi concentration exponentially dropping along the growth direction at first 25 nm Ga(As,Bi) film thickness and then stabilizing at a constant level^31^. In the core–shell NW heterostructures investigated here, the thickness of Ga(As,Bi) shells is in the range of 14–26 nm, hence the same effect can occur. Moreover, as revealed by the Monte Carlo simulations of Bi incorporation and droplets formation during the MBE growth of Ga(As,Bi), once the surface Bi droplets are formed the incorporation of Bi into the growing film decreases considerabely^15^. In our case, both effects can occur. In the cross-section of a ZB NW part, we also observe the enhanced Bi content at the outer NW region close to the sidewall surface and at the outer NW corner.

## Conclusions

In conclusion, we have investigated Bi incorporation into predominantly wurtzite GaAs-(Ga,Al)As-Ga(As,Bi) core–shell nanowires grown by molecular beam epitaxy. At low Bi content of 1% the NWs are smooth, with no additional features at their sidewalls, whereas at the attempted Bi content of 4%, the droplets are formed at the NW sidewalls. At the NW tops Bi merges with the Au catalyst and induces (in the NWs cooled down to room temperature) the phase separation into Au_2_Bi and pure Au, however with undisturbed spherical top nanoparticles shapes. In the cross section of the core-multishell GaAs-(Ga,Al)As-Ga(As,Bi) NWs we have detected radially inhomogeneous distribution of Bi. The enhanced Bi content occurs at the inner interfaces of Ga(As,Bi) shells and on their outer surfaces (NW sidewalls). Additionally, in the WZ (Ga,Al)As shells we have observed, so far unnoticed zigzag-shaped enhanced Al content regions along the lines extending radially out of the hexagonal WZ NW corners. In the NWs with mixed ZB-WZ axial structure the preferential location of the side Bi droplets at the WZ NW sections was evidenced. Such Bi droplets induce the growth of WZ GaAs branches perpendicular to the NW trunks. The catalyzing action of Bi droplets, with preferential location at wurtzite nanowire segments, enables controlled formation of branches in the GaAs NWs with mixed WZ-ZB structure, since the structural change between both GaAs polytypes can be induced by alterations of the NW growth conditions.

## Supplementary Information

Supplementary Information.

## References

[1] Pillai MR, Kim SS, Ho ST, Barnett SA (2000). Growth of In_x_Ga_1-x_As/GaAs heterostructures using Bi as a surfactant. J. Vac. Sci. Technol. B.

[2] Usman M (2013). Impact of alloy disorder on the band structure of compressively strained GaBi_x_As_1−x_. Phys. Rev. B.

[3] Oe K, Okamoto H (1998). New semiconductor alloy GaAs_1-x_Bi_x_ grown by metal organic vapor phase epitaxy. Jpn. J. Appl. Phys..

[4] Tixier S, Adamcyk M, Tiedje T, Francoeur S, Mascarenhas A, Wei P, Schiettekatte F (2003). Molecular beam epitaxy growth of GaAs_1-x_Bi_x_. Appl. Phys. Lett..

[5] Wang S, Lu P (2019). Bismuth-containing alloys and nanostructures. Springer Ser. Mater. Sci..

[6] Okamoto H (2015). Supplemental literature review of binary phase diagrams: Bi-Ga, Bi-Y, Ca-H, Cd-Fe, Cd-Mn, Cr-La, Ge-Ru, H-Li, Mn-Sr, Ni-Sr, Sm-Sn, and Sr-Ti. J. Phase Equilibr. Diffus..

[7] Masnadi-Shirazi M, Lewis RB, Bahrami-Yekta V, Tiedje T, Chicoine M, Servati P (2014). Bandgap and optical absorption edge of GaAs_1−x_Bi_x_ alloys with 0 < x < 17.8%. J. Appl. Phys..

[8] Wang L (2017). Novel dilute bismide, epitaxy, physical properties and device application. Curr. Comput.-Aided Drug Des..

[9] Huang H, Liu J, Duan W (2014). Nontrivial Z2 topology in bismuth-based III–V compounds. Phys. Rev. B.

[10] Hsu C-H (2018). Growth of a predicted two-dimensional topological insulator based on InBi-Si(111)-√7 ×√7. Phys. Rev. B.

[11] Nicolaï, Laurent, *et al*. Topological surface states from ordered InBi crystals. arXiv: 1806.03061 (2018).

[12] Fang Z, Gao H, Venderbos JWF, Rapp AM (2020). Ideal near-dirac triple-point semimetal in III–V semiconductor alloys. Phys. Rev. B.

[13] Caroff P, Bolinsson J, Johansson J (2011). Crystal phases in III–V nanowires: from random toward engineered polytypism. IEEE J. Sel. Top. Quantum Electron..

[14] Lewis RB, Masnadi-Shirazi M, Tiedje T (2012). Growth of high Bi concentration GaAs_1−x_Bi_x_ by molecular beam epitaxy. Appl. Phys. Lett..

[15] Rodriguez GV, Millunchick JM (2016). Predictive modeling of low solubility semiconductor alloys. J. Appl. Phys..

[16] Harmand JC, Tchernycheva M, Patriarche G, Travers L, Glas F, Cirlin G (2007). GaAs nanowires formed by Au-assisted molecular beam epitaxy: Effect of growth temperature. J. Crystal Growth.

[17] Sadowski J (2007). GaAs: Mn nanowires grown by molecular beam epitaxy of (Ga, Mn)As at MnAs segregation conditions. Nano Lett..

[18] Rudolph A (2009). Ferromagnetic GaAs/GaMnAs core-shell nanowires grown by molecular beam epitaxy. Nano Lett..

[19] Siusys A, Sadowski J (2014). All wurtzite (In, Ga)As-(Ga, Mn)As core-shell nanowires. Nano Lett..

[20] Sadowski J (2017). Wurtzite (Ga, Mn)As nanowire shells with ferromagnetic properties. Nanoscale.

[21] Liu Yi (2021). Self-selective formation of ordered 1D and 2D GaBi structures on wurtzite GaAs nanowire surfaces. Nat. Commun..

[22] Laukkanen P, Sadowski J, Guina M, Patane A, Balkan N (2012). Characterization of III-V surfaces with low energy electron diffraction and reflection high-energy-electron diffraction. Semiconductor Research.

[23] Kret S, Kaleta A, Bilska M, Kurowska B, Siusys A, Dabrowski J, Sadowski J (2017). FIB Method of Sectioning of III-V Core-multi-shell nanowires for analysis of core/shell interfaces by high resolution TEM. Acta Phys. Pol..

[24] Panciera F (2020). Phase selection in self-catalyzed GaAs nanowires. Nano Lett..

[25] Jaggi R (1964). Struktur und eigenschaften der hochdruck-modifikation Bi II. Helv. Phys. Acta.

[26] Dubrovskii VG, Sibirev NV, Cirlin GE, Harmand JC, Ustinov VM (2006). Theoretical analysis of the vapor-liquid-solid mechanism of nanowire growth during molecular beam epitaxy. Phys. Rev. B.

[27] Rudolph D (2013). Spontaneous alloy composition ordering in GaAs-AlGaAs core-shell nanowires. Nano Lett..

[28] Mancini L (2014). Three-dimensional nanoscale study of Al segregation and quantum dot formation in GaAs/AlGaAs core-shell nanowires. Appl. Phys. Lett..

[29] Wolf D (2018). Three-dimensional composition and electric potential mapping of III−V core-multishell nanowires by correlative STEM and holographic tomography. Nano Lett..

[30] Zhang Y (2019). Highly strained III-V-V coaxial nanowire quantum wells with strong carrier confinement. ACS Nano.

[31] Reyes DF, Bastiman F, Hunter CJ, Sales DL, Sanchez AM, David JP, González D (2014). Bismuth incorporation and the role of ordering in GaAsBi/GaAs structures. Nanoscale Res. Lett..

