# Supplementary material for: Bi incorporation and segregation in the MBE-grown GaAs-(Ga,Al)As-Ga(As,Bi) core–shell nanowires
Source: Sci Rep. 2022 Apr 9;12:6007. doi: 10.1038/s41598-022-09847-w (PMC8994761; doi:10.1038/s41598-022-09847-w)
Supplement: Supplementary file 1 — Supplementary Information. [file 41598_2022_9847_MOESM1_ESM.docx]

Supplementary material

Bi incorporation and segregation in the MBE-grown GaAs-(Ga,Al)As-Ga(As,Bi) core-shell nanowires

Janusz Sadowski^1,2^, Anna Kaleta^1^, Serhii Kryvyi^1^, Dorota Janaszko^1^, Bogusława Kurowska^1^, Marta Bilska^1^, Tomasz Wojciechowski^1,3^, Jarosław Z. Domagala^1^, Ana M. Sanchez^4^, and Sławomir Kret^1^

^1^ Institute of Physics Polish Academy of Sciences, Aleja Lotnikow 32/46, PL-02668 Warsaw, Poland

^2^ Department of Physics and Electrical Engineering, Linnaeus University, SE-39182 Kalmar, Sweden

^3^ International Research Centre MagTop, Institute of Physics, Polish Academy of Sciences, Aleja Lotnikow 32/46, PL-02668 Warsaw, Poland

^4^ Department of Physics, University of Warwick, Coventry CV4 7AL, United Kingdom

corresponding authors:

[janusz.sadowski@lnu.se](mailto:janusz.sadowski@lnu.se)

kret@ifpan.edu.pl

**1. Planar Ga(As,Bi) layers grown together with GaAs-Ga(As,Bi) core-shell nanowires.**

**1.1. Characterization by X-ray diffraction and SEM**

The planar layer grown on the Au-free GaAs(001) substrate together with sample 1 (GaAs – Ga(As,Bi) core-shell NWs) was measured by high resolution X-ray diffraction (XRD) X’Pert MRD with Cu tube (CuK_α1_ radiation (λ = 1.5406 Å)), equipped with: X-ray mirror, monochromator (asymmetrically cut Ge 4x(220)) and with two proportional detectors, one of the detectors is preceded by the analyzer 3xGe(220).

Figure S1 shows results of 2θ/ω measurement around 004 Bragg reflection of GaAs(001) substrate.

**Figure S1**. X-ray diffraction results - 2θ/ω measurement, 004 Bragg reflection of a planar Ga(As,Bi) layer grown together with the nanowires of sample 1, with Bi effusion cell temperature T_Bi_ = 540 °C.

The perpendicular lattice parameter obtained from the angular position of the peak corresponding to Ga(As,Bi) layer (grown simultaneously with Ga(As,Bi) shells of GaAs NWs on Au-coated GaAs(111)B) equals to: a_⊥_ = 5.7126 Å. The Ga(As,Bi) layer is fully strained to the GaAs substrate, hence we can calculate the relaxed lattice parameter (assuming the Ga(As,Bi) elastic constant values C_11_ and C_12_ to be the same as for GaAs) which amounts to: a_relaxed_ = 5.68473 Å. Taking the hypothetical lattice parameter of binary GaBi equal to 6.33 Å and assuming Vegards law we obtain then 4.6% Bi content in the planar Ga(Bi,As) layer grown together with sample 1. The distance between pendelösung fringes, confirmed by the XRD simulations yields the thickness of Ga(As,Bi) planar layer equal to 137 nm, which agrees quite well with the value assumed from the GaAs growth rate calibrations based on RHEED oscillations.

Apparently, in the planar zinc-blende (ZB) counterpart of sample 1 the incorporation of Bi is much higher than that in the WZ Ga(As,Bi) NW shell. EDS signal for Bi is low and from the average spectra we obtain values at the level of 1at %, however with large error (about the same order of magnitude, i.e. ±1%). Nevertheless, the Bi-M line in the X-ray spectrum is clearly above the noise level. Analysis of different spectra gives similar results, around 1at%.

The surface of the reference planar layer grown together with sample 1 is a bit “milky” (as observed by naked eye), suggesting the presence of some 3D objects (Bi droplets) but it still shows distinct 2D RHEED patterns, hence the concentration of the droplets is not huge. However, for samples 2 and 3, grown with about 4 times higher Bi flux, corresponding to the temperature of Bi source T_Bi_ = 580 °C (40 °C higher than that used during the growth of sample 1), RHEED images for reference planar layers disappeared completely upon the growth of Ga(As,Bi). This indicated the presence of amorphous surface features yielding diffused uniform RHEED background. This can be attributed to liquid Bi droplets (the growth temperature is equal 300 °C which is slightly higher than the Bi melting temperature 270 °C)). These droplets (solidified at room temperature of SEM measurements) are shown in Fig. S2, both in plan view and in cross-sectional sample orientation.


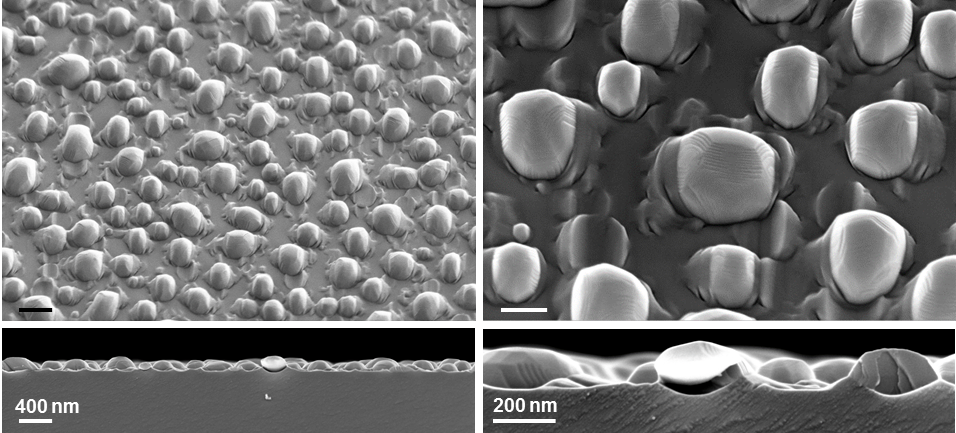


**(a)**

**(c)**

**(b)**

**(d)**

**Figure S2.** SEM images of the surface of Ga(As,Bi)(100) planar layer grown together with Ga(As,Bi)(111) nanowires (sample 2). Panels (a) and (b) - plan view; panels (c) and (d) - cross sectional view of the cleaved sample edge. The scale bars correspond to 400 nm in the panels (a), (c) and to 200 nm in the panels (c) and (d).

**1.2. Characterization by cross-sectional TEM**

More insight into the planar Ga(As,Bi) layer and individual surface Bi droplets is provided by the cross-sectional TEM image of a planar reference to sample 2.


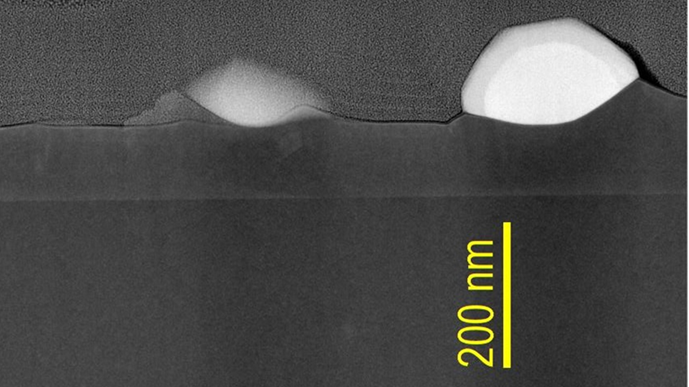


GaAs(100) substrate

Ga(As,Bi) layer

Bi droplets

**Figure S3.** TEM cross-sectional image of Ga(As,Bi)(100) planar layer grown together with Ga(As,Bi)(111) nanowires (sample 2).

Interestingly the surface morphology of planar Ga(As,Bi) in-between segregated Bi droplets is consistent with the predictions based on the Monte-Carlo simulations of Bi droplet segregation during Ga(As,Bi) MBE growth.^[[1]](#endnote-1)^

**2. Supplementary information on nanowires**

Figure S4 shows the top part (a) - ZB, (b) - WZ of the NW taken form sample 2. The oblique stacking fault (SF) defect visible at panel (a) is about 30 deg. inclined vs NW axis, and its end separates two distinct regions of the top droplet - Au_2_Bi and pure Bi. In sample 2 we could identify a lot of NWs with similar top parts.


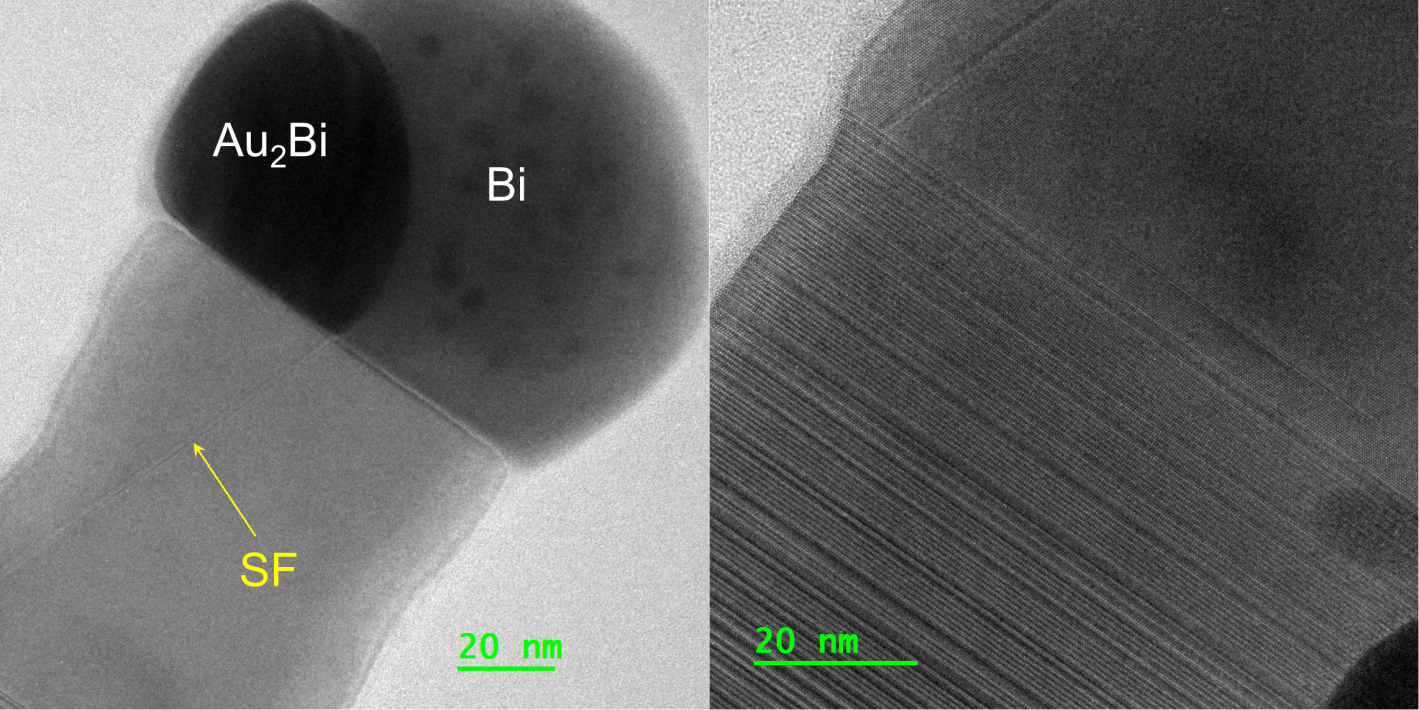


**(a)**

**(b)**

**Figure S4. (**a) - Top (ZB) part of a NW taken from Sample 2 showing inclined stacking fault whose end separates two distinct regions of the top droplet; Au_2_Bi and pure Bi; (b) - The WZ part below the ZB NW neck contains a large number of typical SF defects, perpendicular to the NW axis.


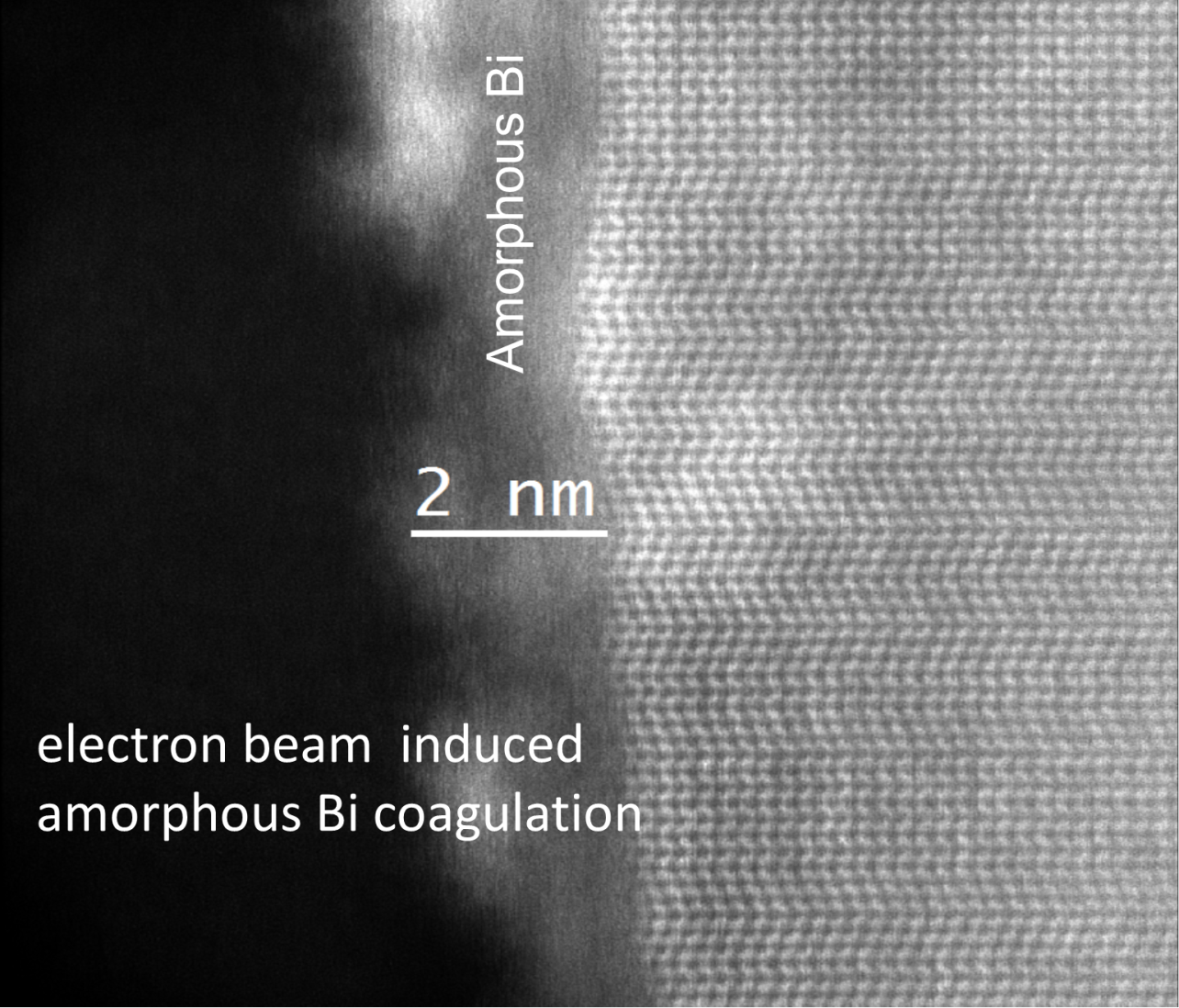


**Fig. S5.** Amorphous Bi “skin” on the sidewall of Sample 2. The Bi amorphization is induced by the e-beam during HRTEM examination.

Figure S.6 shows HRSTEM images revealing the details of the WZ branch generated at WZ NW trunk by the side Bi droplet in the NW taken from sample 3.


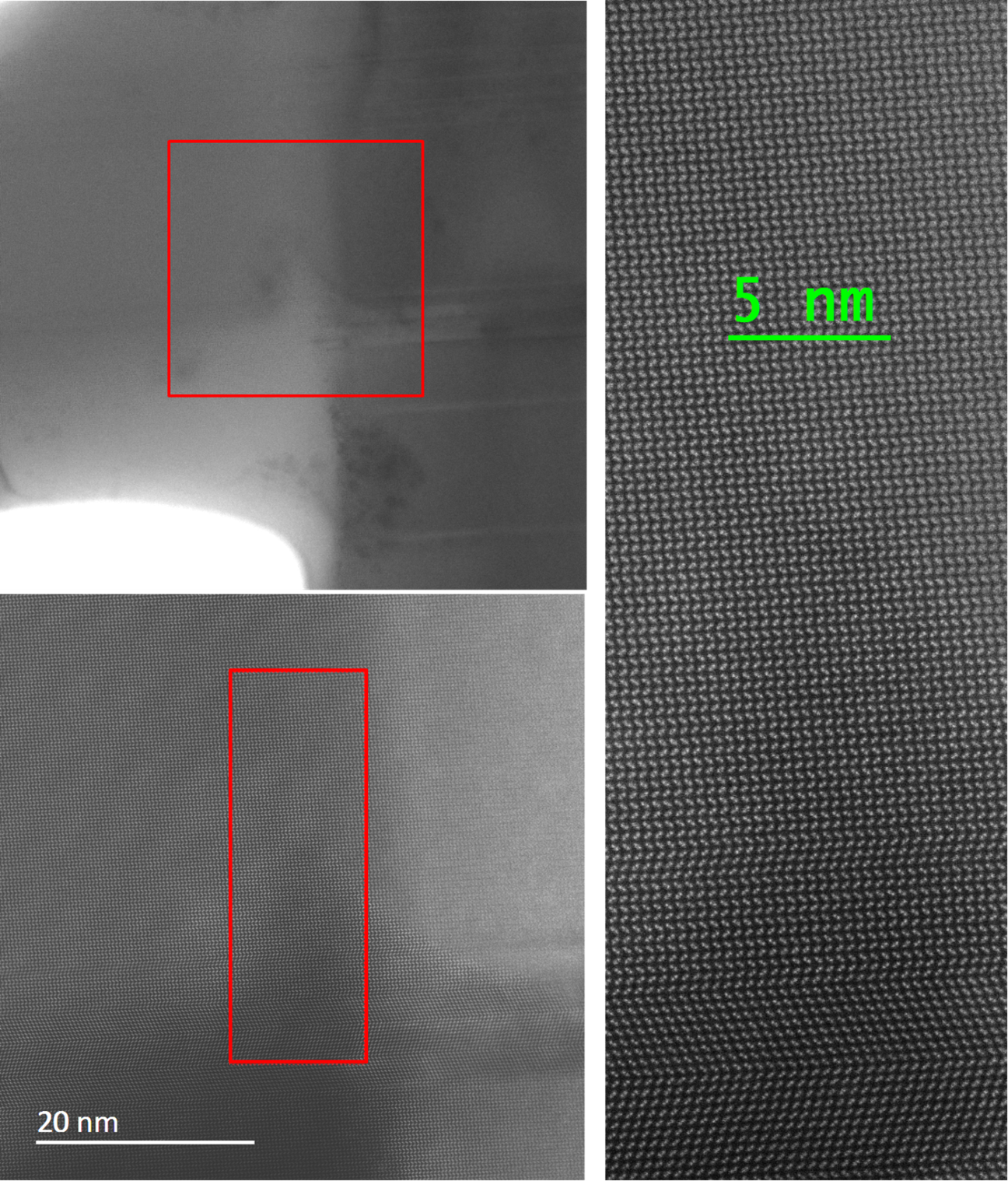


**(d)**

**(c)**


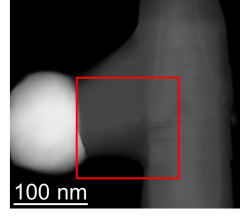


**(a)**

**(b)**

**Figure S6.** STEM image of the WZ GaAs NW branch generated at the WZ GaAs NW trunk (Sample 3). The branch finishes with the catalyzing side Bi droplet. Subsequent panels are magnifications of the regions marked with red rectangles.

**As**


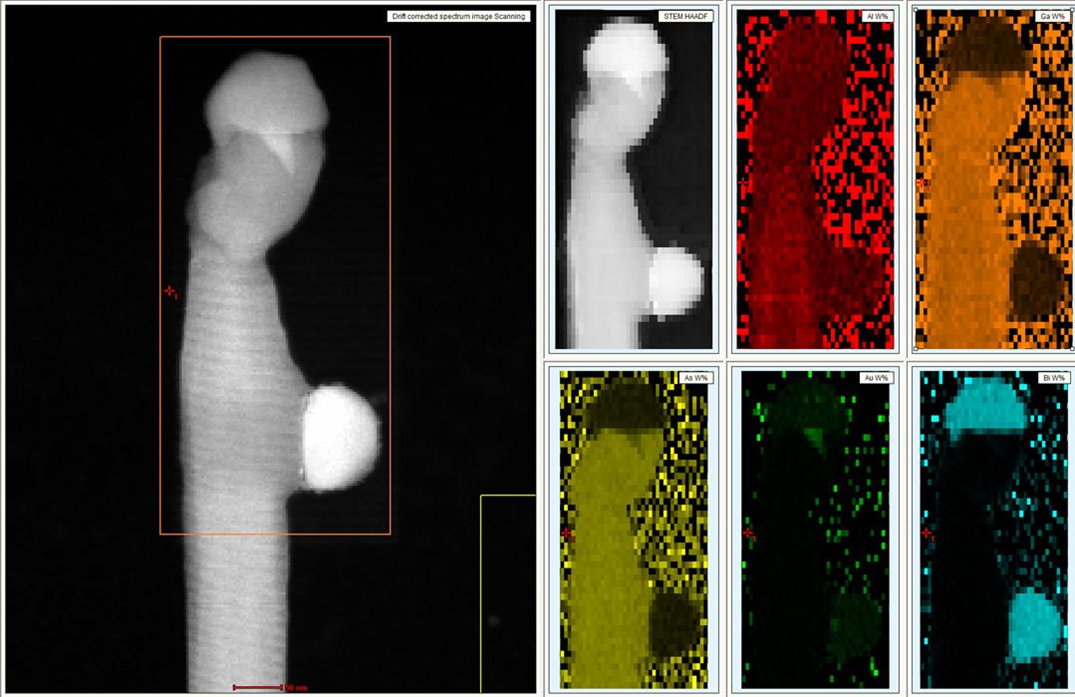


**STEM**

**HAADF**

**Al**

**Ga**

**Bi**

**Au**

**Figure S7.** STEM image (left panel) and EDS composition maps (right panel) of the NW with preserved (unbroken) top part; collected from sample 3.

The STEM images and EDS composition maps of the upper part of the NW collected form sample 3, with unbroken top part are shown in Figure S7. The curved NW part below the very top Bi droplet is axially grown during the Ga(As,Bi) shell deposition and has purely ZB structure,

Figure S8 shows images corresponding to the thin cross-sections of a ZB part of NW selected form sample 3. The thickness of the analyzed NW cross-section is measured using position averaged convergent beam electron diffraction (PACBED) technique. This pattern is obtained using the 9.5 mrad converged electron beam scanned across the core area of the NW cross-section. The comparison of experimental and simulated PACBED patterns is very sensitive to local thickness determination.^[[2]](#endnote-2)^ In our case the best match is measured as *l*^2^-norm metric defined as

$l^{2}\left( t \right)=\sqrt{\sum_{pixels} \left[ \tilde{I}_{exp}-\tilde{I}_{sim}(t) \right]^{2}}$ (1)

Where $\tilde{I}_{exp}$ and $\tilde{I}_{sim}$ denote experimental and simulated PACBED patterns, and t denotes the sample thickness input to the simulation. The minimum difference is obtained for t ≅75 nm. With this specimen thickness only relatively larger composition fluctuations can be determined.


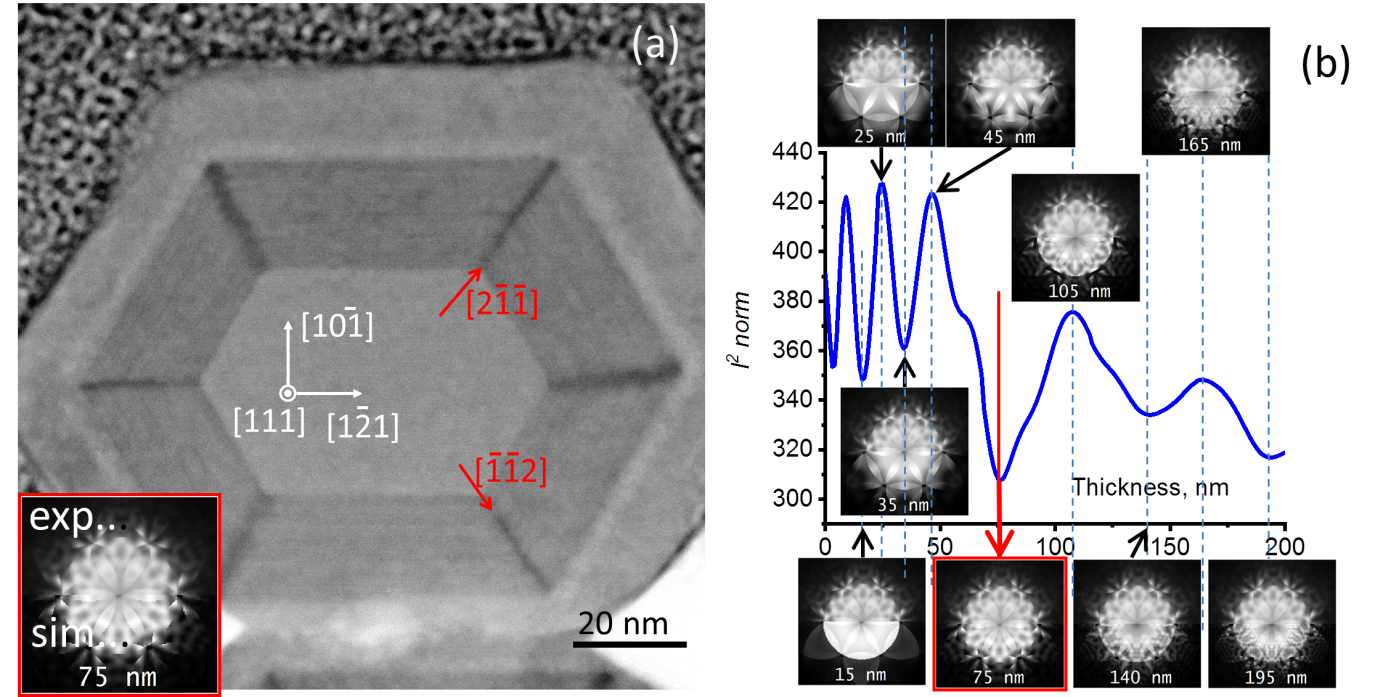


**Figure S8.** Analysis of a thin cross-section of ZB GaAs-(Ga,Al)As-Ga(As,Bi) NW segment (Sample 3). (a) HR-STEM image obtained with the camera length of 73 mm with corresponding best matched PACBED pattern inset; (b) the absolute difference between the experimental and simulated PACBED patterns measured as *l*^2^ -norm metric curve defined by Eq. (1) used for the thickness determination of FIB lamella; the minimum of *l*^2^ –norm is obtained for 75 nm thickness.

To evaluate the impact of defocus on the image contrast viewed in WZ section along [0001] and in ZB one along [111] zone axis we performed series of image simulations, as shown in Fig. S9. From these simulations it is clear that the additional maxima in the middle of the “empty hexagon” of a WZ GaAs crystal structure observed in the [0001] direction cannot appear at any defocus value. Hence the HR-STEM pattern of WZ GaAs in [0001] can always be distinguished from the HR-STEM pattern of ZB GaAs in the [111] zone axis.


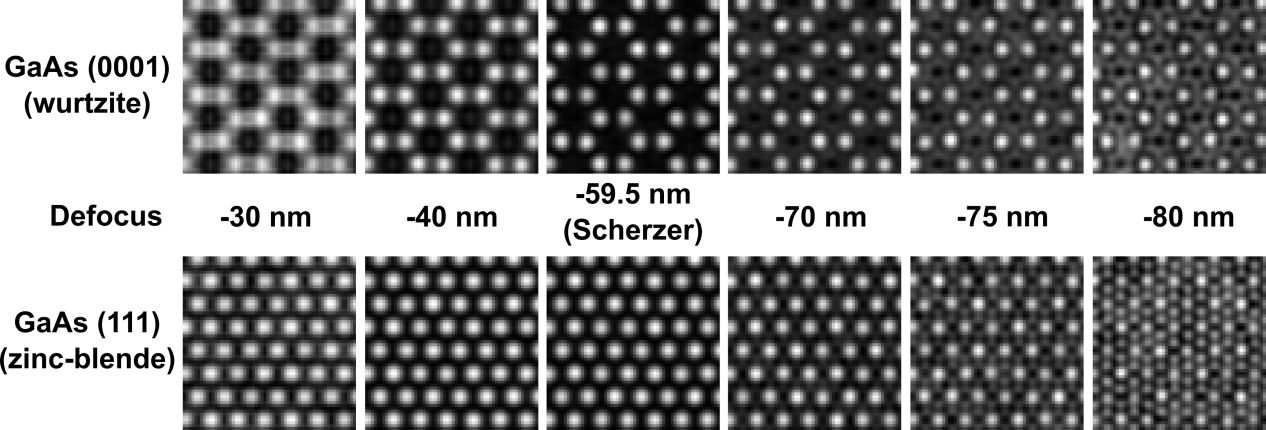


**Figure S9.** Comparison of the HR-STEM simulated data performed for various defocuses for 20 nm thick wurtzite GaAs (0001) and zinc-blende GaAs (111).

The STEM image simulation was carried out using the QSTEM software.^[[3]](#endnote-3)^ The chosen simulation parameters correspond to the experimental conditions used during image acquisition: 300 kV acceleration voltage, 1 eV energy spread of the electron beam, 9.5 mrad convergence angle, spherical aberration coefficient Cs for not aberration corrected of 1.2 mm. The HAADF detector scattering angles range corresponds to the value for 58 mm camera length, that was used for the HR-STEM acquisition. The simulated area for wurtzite GaAs (0001) and zinc-blende GaAs (111) was 15*15 Å with 0.25 Å pixel size.

**References:**

1. . Rodriguez, Garrett, V., and Millunchick, Joanna M. Predictive modeling of low solubility semiconductor alloys. *J. Appl. Phys*. 120, 125310 (2016). [↑](#endnote-ref-1)
2. . Pollock, J. A., Weyland, M., Taplin, D. J., Allen, L. J., Findlay, S. D, Accuracy and precision of thickness determination from position-averaged convergent beam electron diffraction patterns using a single-parameter metric. *Ultramicroscopy* 181, 86 (2017). [↑](#endnote-ref-2)
3. . Koch, C. T. Determination of Core Structure Periodicity and Point Defect Density along Dislocations. *Ph.D. thesis*, Arizona State Univ. 2002. [↑](#endnote-ref-3)
